# Analysis of Slow Wave Oscillations in Cerebral Haemodynamics and Metabolism Following Subarachnoid Haemorrhage

**DOI:** 10.1007/978-1-4939-0620-8_26

**Published:** 2014-03-22

**Authors:** David Highton, Arnab Ghosh, Ilias Tachtsidis, Clare Elwell, Martin Smith

**Affiliations:** 1 Neurocritical Care, University College Hospitals, Queen Square, London, UK; 2 Medical Physics & Bioengineering, University College London, Malet Place, London, UK

**Keywords:** Subarachnoid haemorrhage, Near infrared, Cerebral autoregulation, Ischaemia, Flow-metabolism coupling

## Abstract

Aneurysmal subarachnoid haemorrhage (SAH) causes the greatest loss of productive life years of any form of stroke. Emerging concepts of pathophysiology highlight early abnormalities of microvascular function, including impaired autoregulation of cerebral blood flow and flow-metabolism coupling, as key causes of cerebral ischaemia and poor outcome. Near infrared spectroscopy (NIRS) is a non-invasive optical technique which may help identify cerebral microvascular dysfunction. The aim of this research is to investigate the status of flow-metabolism coupling by examining phase relationships between NIRS-derived concentrations of oxy-haemoglobin ([HbO_2_]), deoxy-haemoglobin ([HHb]) and cytochrome c oxidase oxidation ([oxCCO]). Eight sedated ventilated patients with SAH were investigated. A combined NIRS broadband and frequency domain spectroscopy system was used to measure [HbO_2_], [HHb] and [oxCCO] alongside other multimodal neuromonitoring. Wavelet analysis of phase relationships revealed antiphase [HbO_2_]-[oxCCO] and in-phase [HbO_2_]-[HHb] oscillations between 0.1Hz-0.01Hz consistent with compromised flow-metabolism coupling. NIRS derived variables might offer unique insights into microvascular and metabolic dysfunction following SAH, and in the future identify therapeutic windows or targets.

## Introduction

Aneurysmal subarachnoid haemorrhage (SAH) causes the greatest loss of productive life of all forms of stroke. Only 30 % of patients escape death or major complication [1]. Cerebral aneurysm rupture and extravasation of blood under high pressure leads to immediate and delayed neurological pathology. Emerging evidence highlights the critical role that early abnormalities in microvascular function may contribute to ischaemia; these may manifest as impaired autoregulation of cerebral blood flow (CBF) against blood pressure changes, and deranged flow-metabolism coupling [2].

Animal models of SAH fail to replicate human pathophysiology. Near infrared spectroscopy (NIRS) is a promising non-invasive optical technique which characterises cerebral haemodynamics and metabolism non-invasively, and thus may have widespread applicability investigating human pathophysiology following SAH. NIRS-derived concentration changes of oxy-haemoglobin ([HbO_2_]) and deoxy-haemoglobin ([HHb]) reflect cerebral haemodynamics and may identify impaired pressure autoregulation, associated with vascular dysfunction and ischaemia [3].

Cytochrome c oxidase, the terminal electron acceptor in the mitochondrial respiratory chain reflects the balance between oxygen supply and demand, and its oxidation status [oxCCO] may be measured using NIRS [4, 5]. Intact flow-metabolism coupling results in a characteristic pattern of changes and oscillations in [HbO_2_], [HHb] and [oxCCO] [6]. Typically this results in greater flow than is required by the metabolic demands leading to an increase in [HbO_2_], [oxCCO] and fall in [HHb], a pattern which may be altered in pathology or with variation in flow-metabolism coupling [6, 7]. Slow oscillations (<0.1 Hz) of cerebral haemodynamics and metabolism are seen in neuromonitoring of brain injured patients and the frequency characteristics and phase relationships of these oscillations may be used to characterise cerebral haemodynamics and metabolism.

We hypothesise that the normal [HbO_2_], [HHb] and [oxCCO] phase relationships will be disturbed in SAH patients, indicating impaired flow-metabolism coupling. The aim of this study is to characterise slow oscillations in cerebral haemodynamics and metabolism to investigate microvascular function (cerebral autoregulation and flow-metabolism coupling) within the first 48 h following SAH, where key interventions might be delivered to avoid or minimise ischaemia.

## Methods

Analysis was performed on data from sedated, ventilated patients with SAH, a subset of patients from a larger study investigating [oxCCO] changes in brain injury. Patient characteristics and measured variables were summarised as mean (standard deviation) or median (interquartile range). Data were gathered over a 3-h period, within 48 h of ictus, following institutional Research Ethics Committee approval and representative consent.

Monitoring used for analysis included: invasive arterial blood pressure, transcranial Doppler measured flow velocity in the middle cerebral artery (Vmca; DWL DopplerBox, Compumedics, Germany), brain tissue oxygen tension (PbrO2; Licox, Integra Neurosciences, USA) and NIRS (hybrid optical spectrometer).

The hybrid optical spectrometer comprises two channels capable of simultaneous broadband and frequency domain spectroscopy, optimised for detection of [oxCCO] in brain injury, and has been described in detail elsewhere [4, 8]. [HbO_2_], [HHb] and [oxCCO] were calculated using the UCLn algorithm, fitting NIR attenuation 780–900 nm. The differential pathlength factor (DPF) was calculated from absorption and scattering coefficients derived by the frequency domain system. Only concentration changes measured ipsilateral to invasive monitoring at 35 mm source detector separation was considered.

Transient artefacts were removed by interpolation. Systemic data, PbrO2 and Vmca, were synchronised (using a synchronisation signal at start and finish) and resampled to 1 Hz for analysis (*resample*, Matlab, Mathworks). NIRS data were analysed at its native sampling frequency (0.31 Hz). Autoregulation indices were derived from a moving continuous Pearson correlation coefficient between 30 epochs of 10 s time averaged data between arterial blood pressure and neuromonitoring (Vmca and PbrO_2_), yielding the mean velocity index, and oxygen reactivity index respectively, as surrogate markers of pressure autoregulation and impaired vascular function [9]. These validated indices of autoregulation suggest impaired autoregulation when >0.3. NIRS phase difference and coherence measurements were calculated using a wavelet based approach (complex Morlet wavelet, Matlab, Mathworks) measuring the instantaneous phase difference and wavelet coherence [10] from scales 1 to 100 (frequency 0.3–0.003 Hz).

## Results

Eight patients were studied, their characteristics and autoregulation indices are summarised in Table 26.1 and monitored variables in Table 26.2. Three patients showed evidence of impaired pressure autoregulation indicated by an oxygen reactivity index and/or mean velocity index above 0.3.

**Table 26.1:** Patients characteristics and autoregulation indices

Patient characteristics	
Age in years (range)	50.3 (23–74)
Sex	7 female, 1 male
Median Glasgow Coma Scale (IQR)	5.5 (3–8)
Mean Oxygen reactivity index (SD)	0.03 (0.21)
Mean velocity index (SD)	0.16 (0.15)

**Table 26.2:** Patients monitored variables

Monitored variables	Mean (SD)
Mean arterial pressure (mmHg)	93 (8)
PbrO2 (mmHg)	26 (12)
Vmca (cm/s)	56 (18)

**Fig. 26.1:**
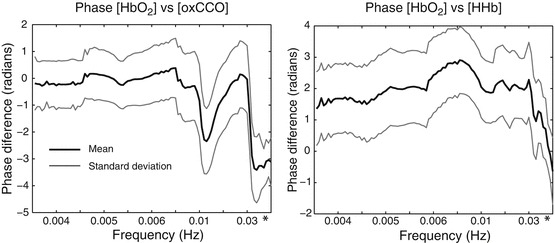
These graphs demonstrate the group phase differences. Specific features are apparent between 0.1 and 0.01 Hz with anti phase [HbO_2_] versus [CCO] activity. The *asterisk* marks 0.1 Hz. The pseudofrequency of wavelet scale is shown resulting in a non-linear x-axis

**Fig. 26.2:**
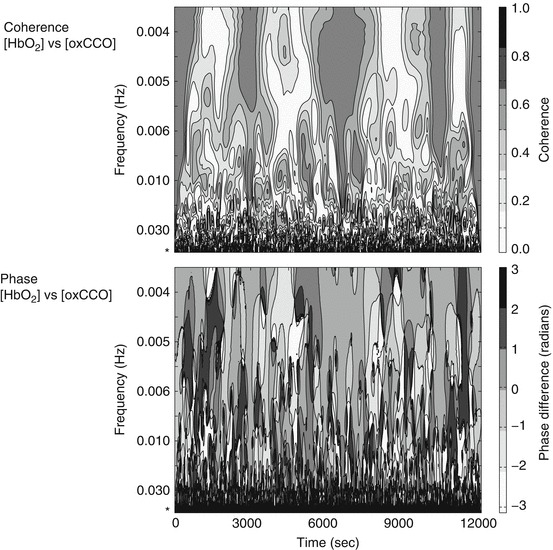
Wavelet coherence and phase difference are shown for [HbO_2_] versus [CCO] in an individual patient. Time is represented on the x axis and a non-linear representation of frequency on the y axis. Again a band of interest can be observed between 0.1 Hz (indicated by *asterisk*) and 0.03 Hz. A band of coherence in this frequency region (*dark grey*) indicates a strong relationship between the signals. The phase difference plot shows similar findings to the group data in this region—around 0.1 Hz [HbO_2_] is antiphase to [CCO] indicated by *black*/*dark-grey*, at 0.01 Hz this changes to predominately *light grey* indicating phase difference close to 0. Some dynamic variation over time can be observed; however, these relationships remain considerably consistent over the 3-h period. The pseudofrequency of wavelet scale is shown resulting in a non-linear y-axis

Phase difference, [HbO_2_] versus [HHb] and [HbO_2_] versus [oxCCO] are displayed in Fig. 26.1, demonstrating a key feature in the band 0.1–0.01 Hz where [oxCCO] approaches being antiphase to [HbO_2_]. In contrast [HHb] is in phase with [HbO_2_] at 0.1 Hz developing a phase lag (towards 1 radian) below 0.02 Hz. The time course of coherence and phase difference in a representative patient are displayed in Fig. 26.2.

## Discussion

We have demonstrated evidence of impaired microvascular control of CBF in this group of critically ill patients with SAH, manifest as: (1) Impaired pressure autoregulation in 3/8 patients, and (2) NIRS phase relationships which suggest impaired flow-metabolism coupling of CBF to energy requirements.

Impaired pressure autoregulation is increasingly described in association with poor outcome following SAH, but it remains unclear whether this is due to ischaemia consequent to impaired autoregulation, or if this impairment is just a symptom of dysfunctional injured cerebral tissue [3]. The phase relationships between NIRS variables at 0.1 Hz are consistent with those observed in experimental models of ischaemia, and cortical spreading depression [10]. [oxCCO] reflects the dynamics of mitochondrial electron transport and presents a unique window into subcellular energetics. The observed occurrence of antiphase oscillations with [HbO_2_] are consistent with suboptimal oxygen delivery in response to metabolic demand; this has previously been observed in animal models of cortical spreading depression [10], but also human functional activation [6, 11]. Crucially ischaemia results from failure of energy supply or utilisation—so the measurement of [oxCCO], a measure of cerebral oxygen utilisation, may provide valuable additional information over and above markers of haemodynamics.

The hybrid optical spectrometer has been specifically optimised for the detection of [oxCCO] in adult brain injury, combining broadband spectroscopy to aid separation of chromophores and frequency domain spectroscopy to calculate DPF. This robust solution reduces concerns that oscillations observed in [oxCCO] might be due to variation in DPF or crosstalk. Importantly we have observed distinct patterns of phase-relationship between [HbO_2_], [HHb] and [oxCCO], and this adds weight to the argument that [oxCCO] is a distinct signal of relevance. However, quantifying oscillations at 0.1 Hz is at the absolute limit of this device as the sampling period of each reading is 3.2 s. Wavelet methods of analysis also trade off between frequency resolution and time resolution, but are superior in that they discriminate important changes in the time domain. Despite these limitations the phase characteristics appear in broad bands, particularly within 0.1–0.01 Hz. Thus, we believe that the NIRS instrumentation and the analysis techniques described are both sufficient to demonstrate the key features of interest.

NIRS oscillations following SAH may reflect impaired autoregulation and flow metabolism coupling—consistent with proposed microvascular dysfunction mediated via nitric oxide or spreading cortical depression [2]. Monitoring the evolution of microvascular dysfunction in the first 48 h following SAH might identify pathological processes that allow for timely and targeted intervention [2]. Further work is required to elucidate the exact pathophysiology underpinning the haemodynamic and metabolic oscillations we have observed, and refine NIRS techniques in the optically complex injured brain. Importantly previous analyses of NIRS oscillations largely reflect vasomotion and haemodynamics [12, 13]. Monitoring [oxCCO] has unique potential to define metabolic compromise in SAH, and might be used in the future to guide neuroprotective strategies.
